# Thyroid Hormone Supplementation Therapy for Differentiated Thyroid Cancer After Lobectomy: 5 Years of Follow-Up

**DOI:** 10.3389/fendo.2020.00520

**Published:** 2020-07-31

**Authors:** Soo Young Kim, Hee Jun Kim, Seok-Mo Kim, Hojin Chang, Yong Sang Lee, Hang-Seok Chang, Cheong Soo Park

**Affiliations:** ^1^Department of Surgery, Thyroid Cancer Center, Gangnam Severance Hospital, Institute of Refractory Thyroid Cancer, Yonsei University College of Medicine, Seoul, South Korea; ^2^Department of Surgery, CHA Ilsan Medical Center, Goyang-si, South Korea

**Keywords:** thyroid stimulating hormone suppression, hypothyroidism, low-risk differentiated thyroid cancer, levothyroxine supplementation, thyroid lobectomy

## Abstract

**Background:** Lobectomy with preservation of the contralateral lobe has already become the most preferred surgical method for patients with low-risk thyroid cancer. The incidence of and risk factors for the development of hypothyroidism after lobectomy for thyroid cancer remains unclear. The previous practice of levothyroxine supplementation post-thyroidectomy, to bring about thyroid stimulating hormone (TSH) suppression, had some serious side effects. This study aimed to evaluate the incidence of hypothyroidism and to identify the factors associated with hypothyroidism requiring thyroid hormone replacement.

**Methods:** We retrospectively reviewed the charts of 256 consecutive patients with differentiated thyroid cancer treated with lobectomy at the Gangnam Severance Hospital between April and December 2014 who were followed-up for more than 5 years. Patients were evaluated using a thyroid function test at the time of outpatient visit every 6 months for the 1st year, with an annual follow-up thereafter.

**Results:** After 5 years, 66.0% (169) of the patients needed levothyroxine supplementation to maintain euthyroid status. The incidence of hypothyroidism requiring levothyroxine supplementation increased until 3 years but showed no significant change in the 4 and 5th year. Recurrence showed no difference between the group with and without levothyroxine supplementation. The presence of thyroiditis and preoperative TSH levels were correlated with postoperative levothyroxine supplementation to maintain euthyroid status, in univariate and multivariate analyses.

**Conclusion:** High preoperative TSH levels and/or thyroiditis indicate a significantly increased likelihood of developing hypothyroidism requiring thyroid hormone supplementation after a thyroid lobectomy. Patients with an increased risk of postoperative hypothyroidism must be aware of their risk factors and should undergo more intensive follow-ups.

## Introduction

With the improvement in early diagnosis, the proportion of patients with low-risk differentiated thyroid cancer (DTC) is also increasing. Lobectomy with preservation of the contralateral lobe has already become the most preferred surgical method for patients with low-risk thyroid cancer (1).

The incidence of and risk factors for the development of hypothyroidism after lobectomy remain unclear. Several studies have demonstrated an incidence of post-thyroidectomy hypothyroidism ranging from 9 to 43% depending on the duration of follow-up evaluation and the definition of hypothyroidism among patients who undergo lobectomy (2–4).

Thyroiditis, preoperative thyroid stimulating hormone (TSH) levels, and positivity for thyroid antibodies have been reported to be the most important risk factors for early and late postoperative hypothyroidism (2, 5, 6).

The American Thyroid Association guidelines recommend TSH levels to be in the mid to lower reference range (0.5–2 mU/L) for low-risk patients who have undergone lobectomy, with continued surveillance for recurrence (1). The purpose of levothyroxine therapy is not only to replace the endogenous thyroid hormone to treat hypothyroidism but also to prevent the relapse or progression of thyroid cancer; furthermore, it plays a central role in papillary thyroid carcinoma (PTC) management after thyroidectomy (5, 7). TSH suppression, resulting in serum TSH levels below the lower limit of the reference range, was proposed as a therapeutic intervention in thyroid cancer, on the assumption that subnormal serum levels of TSH would slow the growth and spread of thyroid cancer cells (8). While TSH suppression improves disease specific survival in high-risk patients, its benefits in low-risk patients is controversial (5, 7).

TSH suppression, brought about by the long-term administration of supraphysiological doses of levothyroxine, could cause some serious side effects including symptoms and signs of hyperthyroidism and impaired psychological, social, and physical quality of life (9, 10). Increased risks of osteoporosis and fractures, particularly in postmenopausal women, have been reported (11). Moreover, adverse effects on the heart including increased cardiovascular morbidity and mortality are known to be associated with TSH suppression (9, 10, 12).

Defining the risks of hypothyroidism is crucial for developing better preoperative counseling and management strategies and follow-up strategies for patients undergoing this lobectomy. Until now, not much is known about which patients should be carefully followed-up and how the interval of follow-up should be modified.

This study was designed to evaluate the incidence and timing of development of hypothyroidism after lobectomy and to analyze the relationship of post-thyroidectomy hypothyroidism with preoperative parameters and histopathological findings.

## Materials and Methods

### Patients

Among the patients who visited the Thyroid Cancer Clinic at Gangnam Severance Hospital, Yonsei University, 256 consecutive patients who underwent lobectomy for low-risk PTC from April 2014 to December 2014 were enrolled. Patients who had an aggressive variant of papillary thyroid cancer or poorly differentiated thyroid cancer were not included. Patients with preoperative hypothyroidism, defined as patients with known diagnosed hypothyroidism, patients receiving preoperative thyroid hormone treatment for any reason, or patients with baseline TSH levels above the upper limit of the normal range at our institution (0.86–4.69 mcIU/mL) were excluded from the study.

The study was carried out in accordance with the principles laid out in the World Medical Association's Declaration of Helsinki, Good Clinical Practice, and associated Korean regulations. This study was approved by the Institutional Review Boards of Gangnam Severance Hospital. Since patients' identities remained undisclosed as data were obtained retrospectively and since informed consent is not mandatory for retrospective studies in Korea, the institutional review board waived the need for informed consent.

A lobectomy was defined as the resection of either the right or left thyroid lobe with preservation of the isthmus and the contralateral thyroid lobe.

All patients were followed-up postoperatively for at least 5 years from 2014 to 2019. Outpatient follow-up was carried out according to a basic routine protocol: (1) outpatient visits every 6 months for the 1st year, with an annual follow-up thereafter, (2) a thyroid function test at every visit, and (3) an annual sonography follow-up.

### Definition of Hypothyroidism

Postoperative hypothyroidism was defined as a serum TSH level greater than the normal range at our institution (0.86–4.69 mcIU/mL). Thyroiditis was defined as either the presence of positive antibodies preoperatively (anti-thyroid peroxidase antibody or anti-thyroglobulin antibody) or if thyroiditis was diagnosed in a pathologic report.

Levothyroxine supplementation was administered to patients with TSH levels higher than the upper limit of the normal range at our institution.

### Statistical Analysis

Descriptive statistics were used to describe the basic characteristics of the two groups. Continuous variables, expressed as mean ± standard deviation (*SD*), were compared using the Student's *t*-test. Pearson's chi-square test, Fisher's exact test, and McNemar's test were used for categorical variables, expressed as numbers and percentages. Univariate and multivariate analyses were performed by logistic regression analyses. All statistical analyses were performed using SPSS version 23.0 for Windows (SPSS Inc., Chicago, IL, USA). In all statistical analyses, a two-tailed *p* < 0.005 was considered statistically significant.

## Results

The clinicopathological characteristics of the patients included in the study are presented in Table 1. Of the 256 included patients, 169 (66.0%) needed levothyroxine supplementation during follow-up. In three patients (1.2%), recurrence was observed. Investigation of the recurrence site revealed that recurrence occurred in different sites in the three patients: contralateral lobe recurrence, early lung metastasis, and lateral lymph node metastasis.

**Table 1:** Baseline characteristics of the patients who underwent lobectomy.

		**Total patients (*n* = 256)**
Age (years, mean ± *SD*)		43.79 ±10.92
Male		52 (20.3%)
Female		204 (79.7%)
Preoperative TSH (mcIU/mL, mean ± *SD*)		1.79 ± 0.94
Tumor size (cm, mean ± *SD*)		0.72 ± 0.46
Thyroiditis	Yes	87 (34.0%)
	No	169 (66.0%)
*N* stage	N0	187 (73.0%)
	N1a	69 (27.0%)
Recurrence		3 (1.2%)
Mean f/u (days, mean ± *SD*)		1998.7 ± 92.5

*TSH, thyroid stimulating hormone; SD, standard deviation; f/u, follow-up*.

**Figure 1:**
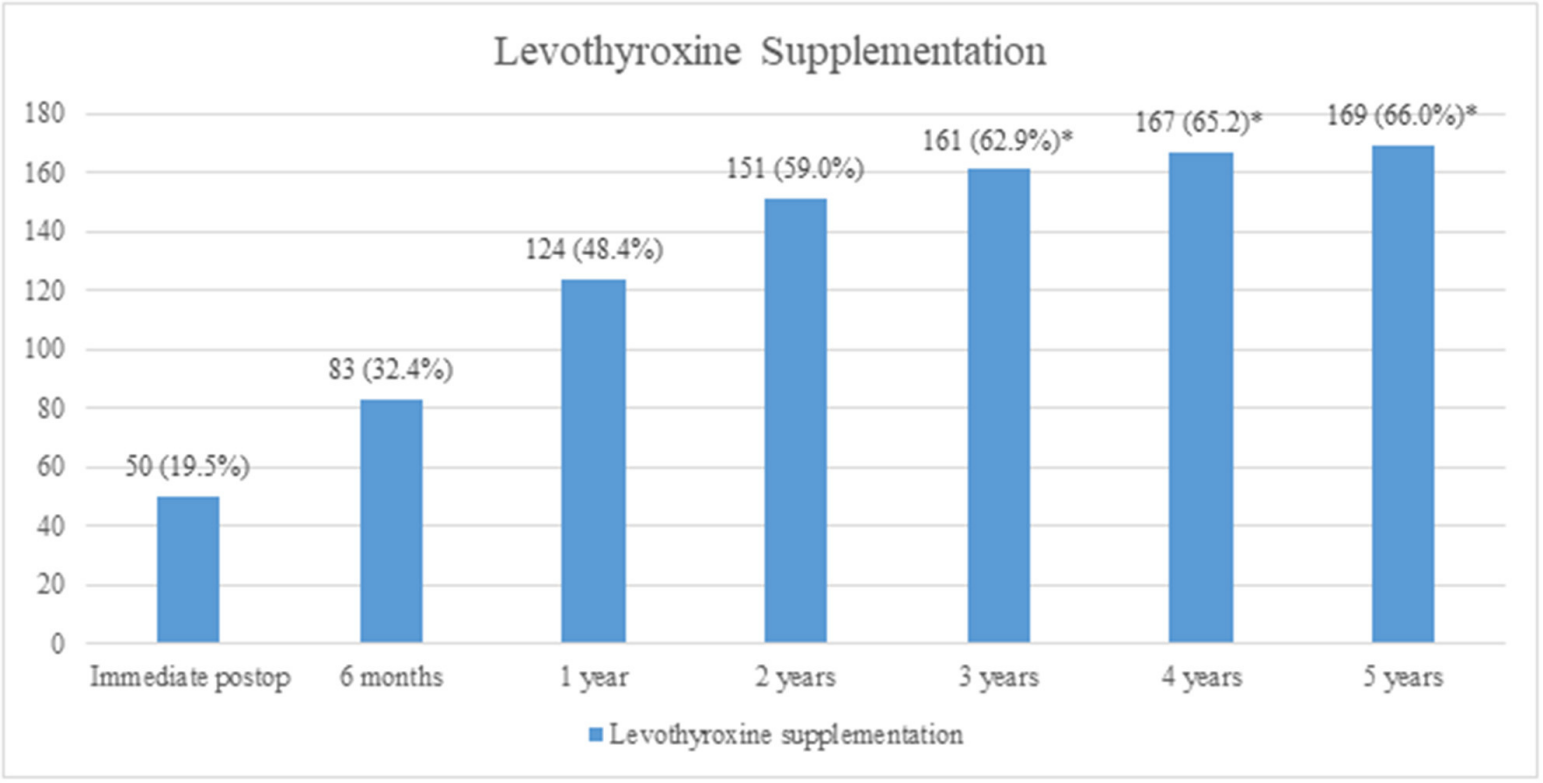
Incidence of hypothyroidism and levothyroxine supplementation. A significant increase in incidence was observed until 3 years (*p* < 0.001), whereas after 3 years, there was no significant increase (*p > 0.05, analyzed by the McNemar's test).

The patients were followed-up for a duration of 5 years. After lobectomy, 19.5% of patients were diagnosed with hypothyroidism immediately postoperatively, and levothyroxine supplementation was started. The incidence of hypothyroidism (with the need of levothyroxine supplementation) increased significantly during the first 3 years of follow-up (*p* < 0.001). After 3 years, there was no significant increase in the incidence of levothyroxine supplementation (Figure 1).

There were no significant differences in sex, tumor size, lymph node metastasis, and recurrence between the groups with and without levothyroxine supplementation. However, age, preoperative TSH levels, and the presence of thyroiditis were significantly different between the two groups (Table 2).

**Table 2:** Clinicopathological characteristics according to levothyroxine supplementation.

	**No levothyroxine (*n* = 87)**	**Levothyroxine (*n* = 169)**	***p*-value**
Age (years, mean ± *SD*)	**43.31 ± 10.21**	**44.04 ± 11.28**	**0.032**
Sex (Female)	70 (80.5%)	134 (79.3%)	0.826
Preoperative TSH (mcIU/mL, mean ± *SD*)	**1.40 ± 0.59**	**1.99 ± 1.02**	**<0.001**
Tumor size (cm, mean ± *SD*)	0.77 ± 0.47	0.69 ± 0.44	0.452
Thyroiditis	**22 (25.3%)**	**65 (38.5%)**	**0.035**
Lymph node metastasis (N1a)	24 (27.6%)	45 (26.6%)	0.870
Recurrence	0 (0%)	3 (1.2%)	0.211

*TSH, thyroid stimulating hormone; SD, standard deviation*.

*Bold values indicates statistically significant different (p < 0.05)*.

In the univariate analysis, there was no correlation between age, sex, and the development of postoperative hypothyroidism, and the need of levothyroxine supplementation. Preoperative TSH levels and the presence of thyroiditis had a significant impact on the incidence of hypothyroidism and levothyroxine supplementation. This was also shown in the multivariate analysis (Table 3).

**Table 3:** Univariate and multivariate analyses for clinical factors associated with levothyroxine supplementation.

	**No levothyroxine (*n* = 87)**	**Levothyroxine (*n* = 169)**	**Univariate**		**Multivariate**	
			**OR (95% CI)**	***p*-value**	**OR (95% CI)**	***p*-value**
Age (years, mean ± *SD*)	43.31 ± 10.21	44.04 ± 11.28	1.006 (0.982–1.030)	0.614	1.002 (0.976–1.028)	0.889
Sex (Female)	70 (80.5%)	134 (79.3%)	1.076 (0.563–2.055)	0.826	1.138 (0.570–2.271)	0.714
Preoperative TSH (mcIU/mL, mean ± *SD*)	1.40 ± 0.59	1.99 ± 1.02	2.229 (1581–3.142)	<0.001	2.271 (1.598–3.227)	<0.001
Thyroiditis	22 (25.3%)	65 (38.5%)	1.847 (1.040–3.279)	0.036	2.021 (1.092–3.738)	0.025

*TSH, thyroid stimulating hormone; SD, standard deviation; OR, odds ratio; CI, confidence interval*.

## Discussion

Our study showed that 66.0% of the patients who underwent lobectomy for PTC developed hypothyroidism and needed levothyroxine supplementation. An increase in the number of hypothyroid patients was observed during the first 3 years of follow-up, but after 3 years, there was no significant increase. The factors associated with hypothyroidism were preoperative TSH levels and thyroiditis. There was no difference in recurrence according to the use of levothyroxine supplementation. Although the patients in this study did not undergo TSH suppression and their TSH levels were maintained in the normal range considered in our hospital, there were only three recurrent cases.

In another study, the authors suggested that they may be able to predict the possibility of developing post-hemithyroidectomy hypothyroidism, especially in the presence of preoperative positivity for microsomal and thyroglobulin antibodies and high-grade lymphocytic infiltration of the resected gland (13). Since we defined “thyroiditis” as the presence of high preoperative levels of antibodies or lymphocytic infiltration in the pathologic report, our study showed the same results. Stoll et al. (14) reported that higher mean preoperative TSH levels, lower preoperative T4 levels, and the presence of Hashimoto's thyroiditis were significant risk factors for hypothyroidism. Specifically, patients with preoperative TSH levels >1.5 uIU/mL had a higher proportion of hypothyroidism. Furthermore, female sex could also be a potential risk factor for hypothyroidism after thyroid lobectomy, although this factor only showed a trend toward statistical significance. However, the above studies only included patients with benign pathology, while patients with malignancy were excluded.

The increase in the number of patients who needed levothyroxine supplementation was significant during the first 3 years, whereas there was no significant increase after 3 years. Therefore, we suggest that after lobectomy, patients should be regularly followed-up within short time intervals of 6–12 months for the first 3 years; after 3 years, the follow-up duration can be widened. One study reported that the majority of patients with hypothyroidism were detected during the first 6 months post-operation, and the authors suggested regular follow-ups for serum TSH testing for at least 12 months (13).

Studies have reported that about 14.3–42.6% of patients require thyroid hormone replacement after lobectomy. However, these studies included patients with benign disease and used higher TSH levels to define hypothyroidism (2, 13, 14).

In our study, there was no TSH suppression, and TSH levels were maintained in the normal range (0.86–4.69 mcIU/mL). However, there was no difference in recurrence depending on the incidence of hypothyroidism, and only three patients showed recurrences in this population during the 5 years of follow-up, suggesting that for low-risk DTC patients, there is no need for TSH suppression, and it is sufficient to maintain their TSH levels within the normal range. The findings of the study by Lee et al. support our findings in that considering the excellent prognosis of low-risk DTC and limitations of the effects of TSH suppression therapy, TSH suppression treatment is not necessary for patients who undergo lobectomy for low-risk DTC (15). Another study reported that serum TSH levels did not affect short-term recurrence in patients with low-risk DTC after thyroid lobectomy. During a 5-years follow-up, 1.4% of patients experienced recurrence. The mean TSH values did not affect recurrence-free survival. However, the mean TSH levels of patients in their study were within the recommended low-normal range (0.5–1.9 mIU/L) (16). Furthermore, a randomized controlled trial with low-risk PTC patients showed that disease-free survival (DFS) in patients without TSH suppression was not inferior by more than 10% to the DFS in patients with TSH suppression. The authors suggested that thyroid-conserving surgery without TSH suppression should be considered for patients with low-risk PTC to avoid the potential adverse effects of TSH suppression (17). However, the follow-up time in our study was only 5 years and required a more prolonged time of observation to follow-up for recurrence.

Research has been conducted not only on the effect of TSH suppression on cancer recurrence but also on its role in the prevention of nodular recurrence of benign disease. It has been reported that prophylactic levothyroxine treatment after lobectomy significantly decreased the recurrence rate of nodular goiter in the contralateral thyroid lobe as well as the need for completion thyroidectomy, mostly among patients with iodine deficiency (18). Levothyroxine therapy may prevent the recurrence of nodular disease; furthermore, levothyroxine therapy at a substitutive dosage may be sufficient compared to TSH suppression (19).

In conclusion, interpretation of these results and related literature has several practical implications in clinical settings. High preoperative TSH levels and/or thyroiditis should alert the clinician to a significantly increased likelihood of hypothyroidism development and the requirement of thyroid hormone supplementation after thyroid lobectomy. Patients with an increased risk of postoperative hypothyroidism must be aware of their risk factors and should undergo more intensive follow-ups. However, two-thirds of patients who undergo lobectomy need levothyroxine supplementation; thus, from a practical standpoint, all patients who undergo lobectomy should be counseled regarding the potential need for lifelong thyroid hormone therapy.

## Data Availability Statement

The raw data supporting the conclusions of this article will be made available by the authors, without undue reservation.

## Ethics Statement

The study was carried out in accordance with the principles laid out in the World Medical Association's Declaration of Helsinki, Good Clinical Practice, and associated Korean regulations. This study was approved by the Institutional Review Board of Gangnam Severance Hospital. As data were obtained retrospectively, patients identities remained, and informed consent is not mandatory for retrospective studies in Korea, the institutional review board waived the need for informed consent.

## Author Contributions

SK, S-MK, HC, H-SC, and CP contributed to the conception and design of the study. SK, HK, and YL organized the database. SK performed the statistical analyses, wrote the first draft of the manuscript, and wrote sections of the manuscript. All authors contributed to manuscript revision and read and approved the submitted version. All authors contributed to the article and approved the submitted version.

## Conflict of Interest

The authors declare that the research was conducted in the absence of any commercial or financial relationships that could be construed as a potential conflict of interest.

## Abbreviations

DFS: Disease-free survival
DTC: Differentiated thyroid cancer
PTC: Papillary thyroid carcinoma
SD: Standard deviation
TSH: Thyroid stimulating hormone
CI: Confidence interval.
